# Neuroprotective and Immunomodulatory Efficacy of Selected Caribbean Medicinal Plants: A Systematic Review

**DOI:** 10.7759/cureus.110137

**Published:** 2026-06-02

**Authors:** Francine F Forbes

**Affiliations:** 1 Behavioral Health and Public Health, Lawrence Medical Center, Lawrence, USA

**Keywords:** annona muricata, caribbean medicinal plants, immunomodulation, momordica charantia, neuroprotection, petiveria alliacea, traditional medicine

## Abstract

The three Caribbean medicinal plants *Momordica charantia* (cerasee/bitter melon), *Annona muricata* (soursop/graviola), and *Petiveria alliacea* (Guinea hen weed/anamu) have been traditionally used to treat inflammatory, metabolic, and neurological disorders. Although research in plant phytochemistry has increased, the evidence base in preclinical settings regarding neuroprotective and immunomodulating effects remains underexplored. This systematic review aimed to synthesize published preclinical evidence on the neuroprotective, immunomodulatory, and anti-inflammatory mechanisms of *M. charantia*, *A. muricata*, and *P. alliacea *and to assess their translational and public health relevance within Caribbean and resource-limited contexts. An exhaustive literature search was performed in PubMed, Scopus, Web of Science, and Cochrane Library through specific Boolean search strategies. Relevant articles were selected based on predefined inclusion criteria by one independent reviewer. Articles that met the inclusion criteria were defined as those consisting of original experimental studies (in vitro, in vivo, or both) involving mechanistic immunomodulatory, anti-inflammatory, and neuroprotective mechanisms. Systematic reviews and articles not containing any mechanistic information were ruled out. The methodological quality of the included papers was evaluated using the MMAT (McGill University, Montreal, Canada) and the JBI critical appraisal tool (JBI, Adelaide, Australia). Since the included studies were diverse, results were described descriptively. Out of a total of 1,182 sources, 13 articles were found to fulfill the criteria: nine on *M. charantia*, two on *A. muricata*, and two on *P. alliacea*. All three medicinal plants demonstrated similar anti-inflammatory effects: downregulation of pro-inflammatory cytokines, inhibition of ROS production, and regulation of key signaling cascades, including PI3K/AKT/NF-κB, MAPK, Nrf2/HO-1, and SIRT1/β-catenin. Neuroprotection included inhibition of neuronal apoptosis, promotion of neural stem cell differentiation, and increased permeability of the blood-brain barrier. Additionally, microbiome alteration and miRNA modulation were recognized as promising mechanisms. Dose-dependent cytotoxic effects were reported for certain compounds, while significant methodology heterogeneity hampered comparability between studies. The reviewed literature supports significant neuroprotective and immunomodulatory effects of these Caribbean medicinal herbs in preclinical settings. Nevertheless, translational research is needed to develop recommendations for the clinical use of these herbs, including pharmacological research and clinical trials.

## Introduction and background

The Caribbean region represents a unique case in global public health, being among the most disaster-prone regions yet among the least prepared to cope with long-term disruptions to the medication distribution chain and health infrastructure [1]. Natural disasters, such as hurricanes, floods, and earthquakes, frequently disrupt access to essential medicines, making community-based health services essential during these periods [2,3]. The increasing interest in indigenous and traditional medicine in this regard stems from the area's inherent vulnerability to prolonged crises, in which external supply cannot be assured [4,5].

Caribbean traditional medicine relies on a diverse ethnobotanical background, influenced by African, Indigenous Amerindian, and European cultures [6]. For instance, *Momordica charantia* (cerasee/bitter melon), *Annona muricata* (soursop/graviola), and *Petiveria alliacea* (Guinea hen weed/anamu) are frequently used to treat inflammatory and fever-related conditions, pain, and metabolic issues [7]. Despite this existing historical pattern of utilization, the scientific basis for their mechanisms of action remains largely limited to preclinical studies, with no synthesis of evidence for these three plant species regarding their neuroprotective and immunomodulatory effects.

The global impetus to integrate traditional medicine into healthcare systems is growing. The World Health Organization (WHO) has published a series of guidelines within the WHO Traditional Medicine Strategy 2014-2023 and an extended version of this strategy through 2023, which call for the creation of evidence-informed policies regarding the role of traditional medicine in achieving Universal Health Coverage and SDG3 [4,8]. Regional frameworks, such as those issued by the Pan-American Health Organization (PAHO) and the Caribbean Public Health Agency (CARPHA), likewise note the capacity for traditional and complementary medicine to play a significant role in building resilient, people-centered health systems, especially in small island developing states that experience recurring outside shocks [9,10].

From a pharmacological perspective, these three medicinal plants have demonstrated similar profiles of bioactive substances active across several neurological and inflammatory pathways. These include polysaccharides such as MCP and MCPS-3; proteins, including MAP30 and α-momorcharin; momordicine; and other bitter melon peptides in *M. charantia* [11,12]. The main bioactive components of *A. muricata* are acetogenins, phenolics, flavonoids, and dietary fibers [13,14]. The two active components identified in *P. alliacea* are the dibenzyl trisulfide (DTS) and the aqueous fractions [15,16], which demonstrate immunomodulatory activity. Preliminary data indicate that these components can modulate oxidative stress, regulate the cytokine network, and influence neuronal survival; however, there is a lack of systematic analysis of their activities.

Three reasons justify choosing these particular plants for the review. First, these plants are the most commonly mentioned medicinal species in the anglophone and wider Caribbean literature. Second, while interest among phytochemists and nutritionists in these plants is increasing, there has been no systematic prior analysis of their neurological and immune effects. Third, the familiarity and availability of these herbs within Caribbean cultures make them ideal candidates for analysis of evidence of the integration of traditional medicine into healthcare in the Caribbean islands.

Accordingly, the purpose of this systematic review is to consolidate the current literature on the neuroprotective and immunomodulatory activities of *M. charantia*, *A. muricata*, and *P. alliacea* in a preclinical setting; elucidate the major signaling pathways responsible for mediating these activities; assess the methodological quality of the studies reviewed; and explore the potential barriers to implementation within a Caribbean health care system framework.

## Review

Methodology

This systematic review was conducted and reported in compliance with the Preferred Reporting Items for Systematic Reviews and Meta-Analyses (PRISMA) 2020 statement. The PRISMA 2020 flow chart was created according to the protocol suggested by Haddaway et al. [17]. Registration in PROSPERO (CRD420261392532) was performed before data collection for this review. Transparency in methodology was ensured by clearly presenting the search strategy, inclusion criteria, and quality assessment steps.

Search Strategy

Systematic searches were performed within four bibliographic databases: PubMed, Scopus, Web of Science, and Cochrane Library. Search terms combined specific plants with outcomes using Boolean operators; search strategies are presented in Table 1. The search was conducted irrespective of publication year.

**Table 1:** Database search strings and initial yields

Database	Search string	Records retrieved
PubMed	(("*Momordica charantia*"[Mesh] OR *Momordica charantia*[Title/Abstract] OR cerasee[Title/Abstract] OR "bitter melon"[Title/Abstract]) OR (*Annona muricata*[Title/Abstract] OR soursop[Title/Abstract] OR graviola[Title/Abstract]) OR (*Petiveria alliacea*[Title/Abstract] OR "guinea hen weed"[Title/Abstract] OR anamu[Title/Abstract])) AND (neuroprotect*[Title/Abstract] OR neuroinflamm*[Title/Abstract] OR "oxidative stress"[Title/Abstract] OR immunomodulat*[Title/Abstract] OR anti-inflammatory[Title/Abstract] OR cytokines[Title/Abstract] OR "immune response"[Title/Abstract] OR "central nervous system"[Title/Abstract])	235
Cochrane Library	((*Momordica charantia* OR cerasee OR "bitter melon"):ti,ab,kw OR (*Annona muricata* OR soursop OR graviola):ti,ab,kw OR (*Petiveria alliacea* OR "guinea hen weed" OR anamu):ti,ab,kw) AND (neuroprotect*:ti,ab,kw OR "oxidative stress":ti,ab,kw OR immunomodulat*:ti,ab,kw OR anti-inflammatory:ti,ab,kw OR cytokines:ti,ab,kw)	3
Scopus	TITLE-ABS-KEY(((*Momordica charantia* OR cerasee OR "bitter melon") OR (*Annona muricata* OR soursop OR graviola) OR (*Petiveria alliacea* OR "guinea hen weed" OR anamu)) AND (neuroprotect* OR neuroinflamm* OR "oxidative stress" OR immunomodulat* OR "anti-inflammatory" OR cytokines OR "immune response"))	190
Web of Science	TS=(((*Momordica charantia* OR cerasee OR "bitter melon") OR (*Annona muricata* OR soursop OR graviola) OR (*Petiveria alliacea* OR "guinea hen weed" OR anamu)) AND (neuroprotect* OR neuroinflamm* OR "oxidative stress" OR immunomodulat* OR "anti-inflammatory" OR cytokines OR "immune response"))	754
Total		1,182

Study Selection and Eligibility Criteria

**Figure 1:**
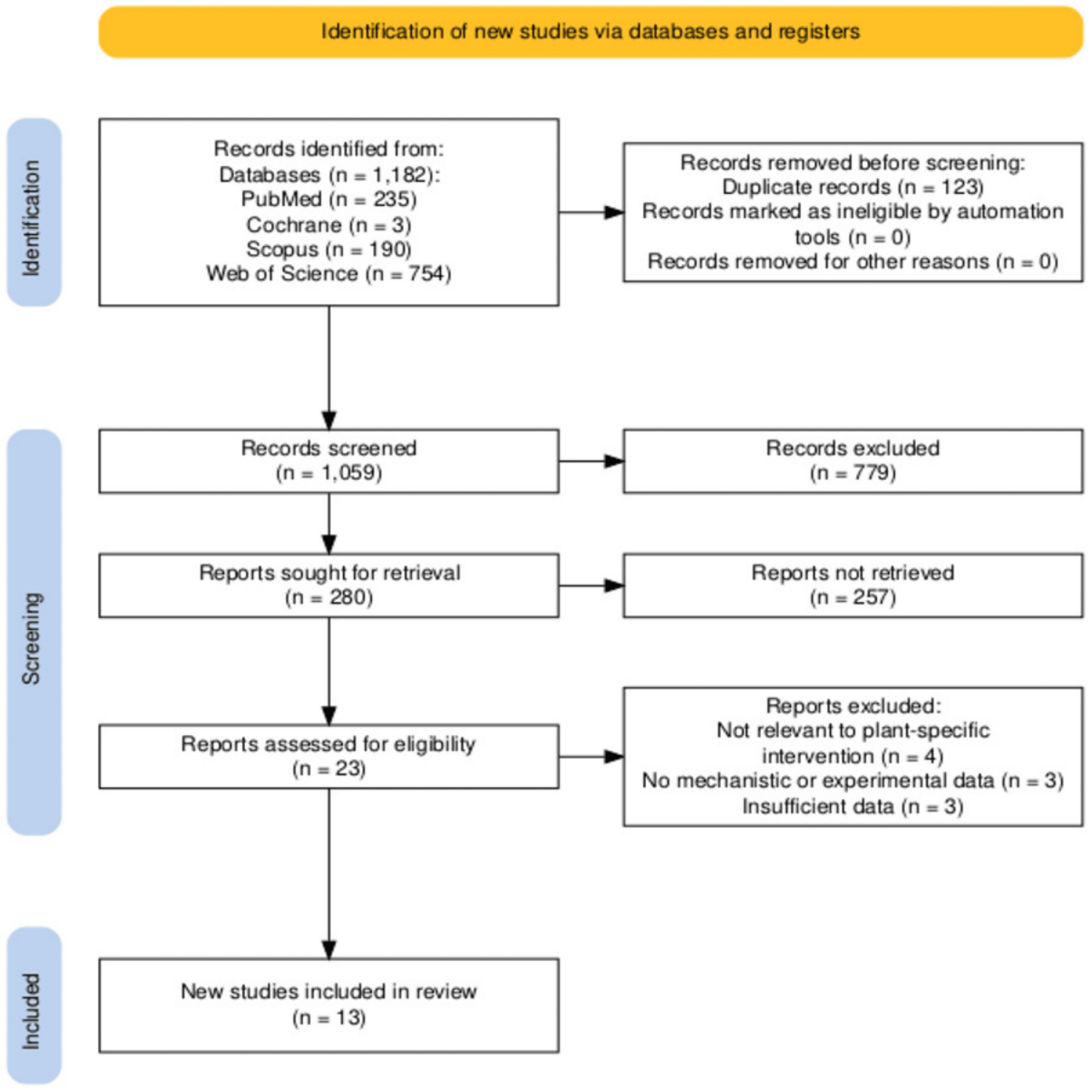
PRISMA 2020 flow diagram of study selection PRISMA: Preferred Reporting Items for Systematic Reviews and Meta-Analyses

The study inclusion procedure is illustrated by the PRISMA 2020 flowchart (Figure 1). After excluding 123 duplicate studies, a total of 1,059 records were screened for their titles and abstracts. Studies were considered eligible if they (1) employed in vitro, in vivo, or integrated animal experimentation; (2) utilized plant extracts, fractions, or molecules extracted from *M. charantia*, *A. muricata*, and/or *P. alliacea*; and (3) provided mechanistic results related to immunomodulation, anti-inflammation, or neuroprotection. Review papers, articles not employing primary experimental approaches, research papers using non-plant intervention treatments, and research that focused solely on cancer-related outcomes or metabolic disorders without mentioning immune and neurological mechanisms were considered ineligible. After the screening phase, 779 records were discarded, and the remaining 280 were reviewed in full. Out of 280 records, 257 reports were not retrieved. In the full-text assessment, 10 of 23 studies were excluded for lacking plant-based experiments, providing inadequate mechanistic outcomes, or relying on secondary data sources.

Data Extraction

Data extraction was performed by a single reviewer using a predefined data extraction framework that included the first author and publication year, country, research methodology, plant species, extract preparation method, model systems, sample size, outcome categories, important outcomes reported, and mechanisms involved in signaling processes. Because a single reviewer performed data extraction and selection, inter-rater reliability was not computed, which was identified as an important methodological limitation. Meta-analysis could not be performed due to substantial heterogeneity among studies in study design, plant preparation methods, models used, and reported outcome measures. A meta-analysis was not conducted due to substantial heterogeneity among the studies in question regarding methodology, plant types and preparations, cell cultures and animal models, measurements taken, and means of presentation, which precluded combining their data and statistically comparing their results.

Quality Appraisal

The methodological quality of the selected literature was evaluated using two separate tools, each adapted to the design of the study included in the synthesis. The MMAT tool (McGill University, Montreal, Canada) was used to evaluate the methodological quality of both in vitro and mixed-methods studies with respect to the clear definition of the research problem, the appropriateness of the methodology, methodological rigor, the validity of the outcomes, the risk of bias, data analysis, interpretability, and reproducibility. On the other hand, the JBI critical appraisal tool (JBI, Adelaide, Australia) was used for in vivo and highly experimental designs, with a focus on the inclusion of randomized control groups, blinding, and mechanistic details.

Characteristics of included studies

The selected studies were published between 2001 and 2026, mostly in Asia (China, Taiwan, and Korea). Still, other countries conducted investigations, including Europe (Germany, Italy, Portugal), Africa (Mauritius, Benin), and South America (Colombia, Brazil). Research design was classified as either entirely in vitro (n = 7), in vivo (n = 3), or both in vitro and in vivo (n = 3) studies. *M. charantia* was the plant most frequently investigated with the most frequently investigated anticancer effects (nine studies). *P. alliacea* and *A. muricata* appeared in two studies each. The types of extracts that were used were highly diverse, including various aqueous and methanolic fractions, polysaccharides, ribosome-inactivating protein (α-momorcharin), bioactive peptides, DTS, and nanoformulated extracts (Table 2).

**Table 2:** Characteristics of included studies *M. charantia*: *Momordica charantia*, *P. alliacea*: *Petiveria alliacea*, *A. muricata*: *Annona muricata*, PMNs: polymorphonuclear neutrophils, WBGE: wild bitter gourd extract, MCAO: middle cerebral artery occlusion, NSCs: neural stem cells, PBMCs: peripheral blood mononuclear cells, MCEE: *M. charantia* ethanol extract, AF: aqueous fraction, DTS: dibenzyl trisulfide, BBB: blood-brain barrier, THP-1: human monocytic leukemia cell line, RAW264.7: murine macrophage cell line

Authors (year)	Country	Design	Plant	Extract type	Model/sample	Sample size
Mahomoodally et al. 2012 [18]	Mauritius/Pakistan	In vitro	M. charantia	Aqueous and methanol fruit extracts	Human blood, PMNs, and mouse monocytes	Not reported
Sung et al. 2018 [19]	Taiwan	In vitro + in vivo	M. charantia	Fruit extract (WBGE)	A549 cells + mice	Not reported
Hu et al. 2020 [20]	China	In vivo + in vitro	M. charantia	Polysaccharides (MCPs)	MCAO rats + NSCs	n = 6/group
Zhang et al. 2025 [21]	China	In vitro fermentation + cell assay	M. charantia	Polysaccharide (MCPS-3)	RAW264.7 macrophages	Not reported
Fachinan et al. 2017 [22]	Benin	In vitro human cell	M. charantia	Fruit and leaf juices	PBMC-derived T cells	Not clearly reported
Kim et al. 2018 [23]	Korea	In vitro	M. charantia	Ethanol extract (MCEE)	SK-N-MC cells	n = 3 experiments
Santander et al. 2012 [24]	Colombia/France	In vitro human cell	P. alliacea	AF	Human dendritic cells	Not clearly reported
Rosner et al. 2001 [25]	Germany	In vitro cell/tissue	P. alliacea	DTS	Neuroblastoma + explants	Not clearly reported
Mancini et al. 2018 [26]	Italy/Portugal	In vitro formulation	A. muricata	Nanoformulated extract	BBB model cells	Not reported
Chuang et al. 2020 [27]	Taiwan	In vitro + in vivo	M. charantia	Methanolic extract	THP-1 + mouse model	n = 5-6/group
Deng et al. 2019 [28]	China	In vivo + in vitro	M. charantia	α-momorcharin protein	Rats + immune cells	Not clearly reported
Leivas et al. 2023 [29]	Brazil	In vivo	A. muricata	Polysaccharide fibers	Mouse model	Not reported
Wu et al. 2026 [30]	China	In vivo + in vitro	M. charantia	Bitter gourd peptides	Lupus mice + macrophages	Not reported

Plant-specific evidence synthesis

**Figure 2:**
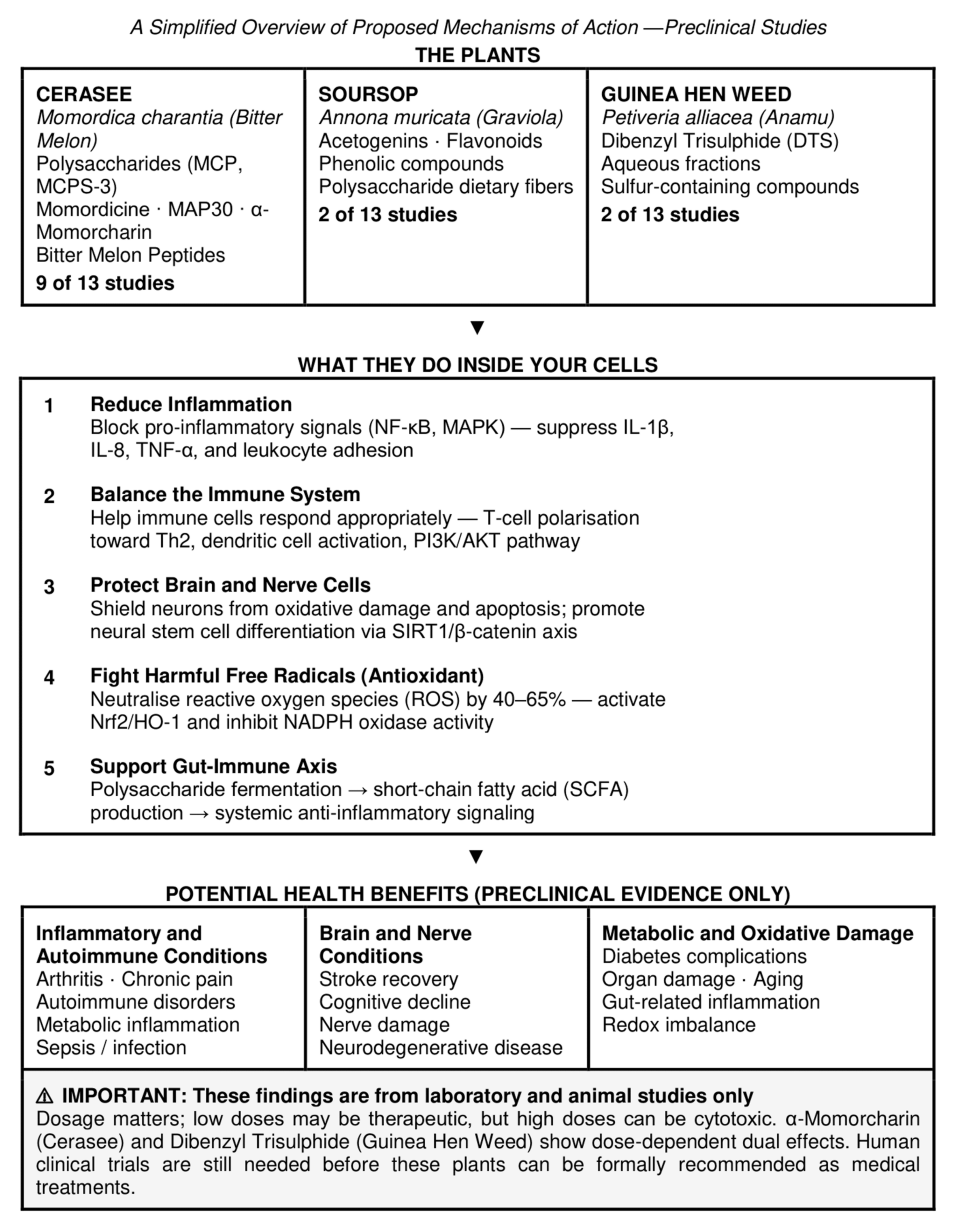
Proposed mechanisms of action of Caribbean indigenous medicinal plants Schematic summarizing the key bioactive compounds, signaling pathways, and therapeutic outcomes identified across 13 preclinical studies. Safety note: dose-dependent dual effects have been documented for α-momorcharin and DTS; low doses exhibit therapeutic activity, whereas higher doses may be cytotoxic. Based solely on preclinical evidence, human clinical data are not yet available for these indications [19-30]. DTS: dibenzyl trisulfide

**Figure 3:**
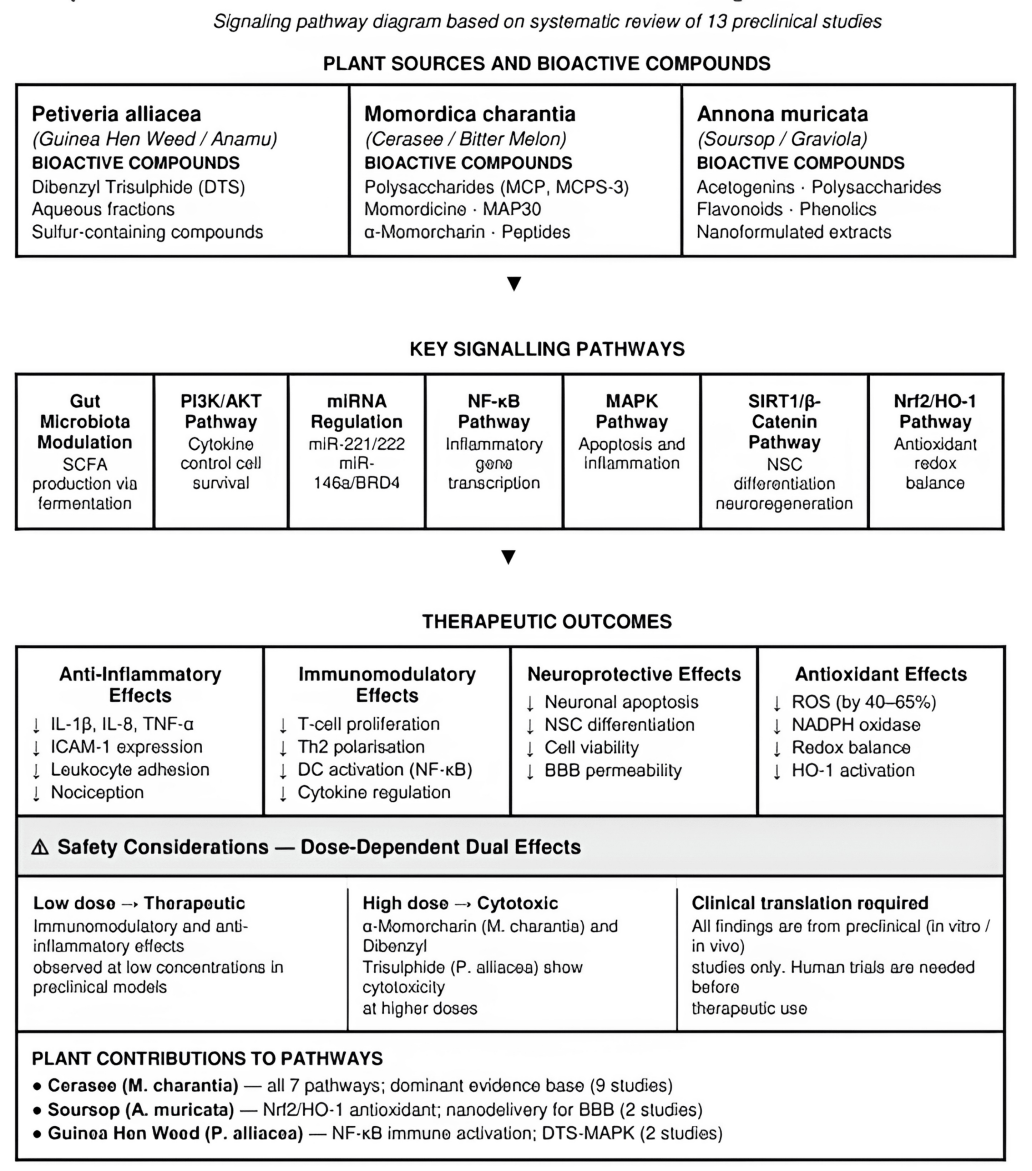
How Caribbean indigenous medicinal plants works in human body Final synthesis includes 13 studies: nine on *M. charantia*, two on *A. muricata*, and two on *P. alliacea*. [4,11,12,31].

Considering the features of the included articles, Figure 2 illustrates the mechanism of action of Caribbean indigenous medicinal plants, based on preclinical study data under review. This illustration includes the main active components, the signaling pathways responsible for their actions, and the therapeutic effects of Caribbean indigenous medicinal plants, including anticancer, antioxidant, anti-inflammatory, and immunomodulating properties. Furthermore, Figure 3 depicts the proposed biological effects of Caribbean indigenous medicinal plants on human health and shows that *M. charantia* is the most commonly reported plant in the included articles, while *P. alliacea* and *A. muricata* are the other two species.

Momordica charantia (Cerasee/Bitter Melon)

Nine of the 13 included studies focus on the anti-inflammatory activities of *M. charantia*, which is more extensively investigated than any other plant included in this review. There are nine different formulations of *M. charantia* extract used in these studies, including aqueous and methanolic fractions, polysaccharides (MCP, MCPS-3), α-momorcharin protein, ethanol extracts, and bioactive bitter gourd peptides. This wide variety of extract forms makes it difficult to compare results across studies but also indicates the diversity of pharmacologically active compounds in this plant.

*M. charantia* has been most strongly validated in the anti-inflammatory domain. Chuang et al. [27] demonstrated that methanolic extract from the leaves of this plant inhibits IL-1β and IL-8 release by inhibiting MAPK and caspase-1 pathways in macrophages and in vivo in a mouse model of cutaneous inflammation. Mahomoodally et al. [18] found that aqueous and methanolic extracts from the fruit of *M. charantia* diminished reactive oxygen species (ROS) by 40-65% by inhibition of nicotinamide adenine dinucleotide phosphate (NADPH) oxidase activity in human immune cells. Microbiota-dependent inhibition of inflammation was demonstrated by Zhang et al. [21], who found that the fermented MCPS-3 fraction produced short-chain fatty acids during simulated gut digestion, thereby downregulating pro-inflammatory cytokine expression in macrophages. The role of *M. charantia* polysaccharides in gut-immune axis modulation is thus expanded to effects occurring within the digestive tract.

Immunomodulatory effects are described in three additional papers. According to Sung et al. [19], wild bitter gourd fruit extract decreased ICAM-1 and TNF-α expression, as well as leukocyte adhesion, via the PI3K/AKT/NF-κB signaling pathway and upstream miR-221/222 regulation, which can be considered one of the most mechanistically clear results in this review. Fachinan et al. [22] observed inhibition of T-cell proliferation and Th2 cell polarization by *M. charantia* fruit juice in peripheral blood mononuclear cell (PBMC)-derived T-cells, suggesting immunosuppressive potential and positioning this plant as a candidate for treating inflammatory and autoimmune diseases. Wu et al. [30] indicated that bitter gourd peptides could inhibit the development of systemic lupus erythematosus by regulating the miR-146a/BRD4 axis in macrophages, highlighting the emerging field of epigenetic immunomodulation.

The neuroprotective properties of *M. charantia* are most convincingly elucidated by two studies. Hu et al. [20] reported that the MCPs stimulated NSC differentiation towards neurons rather than glial cells in the rat model of cerebral ischemia-reperfusion injury by activating the SIRT1/β-catenin pathway. This observation is especially noteworthy because neuroregeneration, unlike neuroprotection, actively influences NSC differentiation. In their work, Kim et al. [23] demonstrated that the ethanol extract inhibited H₂O₂-induced oxidative stress and neuronal apoptosis in SK-N-MC cells by inducing HO-1 and inhibiting MAPK signaling. Deng et al. [28] investigated one of the ribosome-inactivating proteins of *M. charantia*, α-momorcharin. They revealed that its activity exhibited a dual dose-dependent response: at lower concentrations, it downregulated pro-inflammatory cytokine expression, whereas at higher concentrations, it induced apoptosis in monocytes.

Annona muricata (Soursop/Graviola)

Two articles are included for their relevance to distinct aspects of *A. muricata*'s anti-inflammatory and neuroprotective potential. In particular, Mancini et al. [26] encapsulated various formulations of phenolics-enriched aqueous leaf extract in liposomes and phytosomes. They studied their ability to penetrate an in vitro blood-brain barrier (BBB) model. Nanoformulated extract showed increased permeability across the BBB and retained antioxidant activity compared to non-formulated extract. While this paper does not address the anti-inflammatory and neuroprotective mechanisms associated with *A. muricata* leaves, the data are highly translational and indicate that the drug substance can cross the BBB via nanoformulations.

In another article, Leivas et al. [29] studied polysaccharides obtained from *A. muricata* pulp. They demonstrated their analgesic and anti-inflammatory effects by inhibiting the nociceptive response and preventing leukocyte migration. Although the exact mechanisms of action remain unclear, the structure of the polysaccharides, along with anti-inflammatory effects, provides strong biological plausibility for their effects on immune cell migration. The two *A. muricata* papers provide complementary lines of investigation: the first paper demonstrates CNS bioavailability through formulation science, whereas the second paper demonstrates systemic anti-inflammatory and analgesic activity through polysaccharide fractions. Although both are good pieces of scientific research in their respective areas, the mechanistic understanding of this plant is weaker than that of *M. charantia*, and further mechanistic investigation is needed.

Petiveria alliacea (Guinea Hen Weed/Anamu)

This herb appears twice in the literature review, in papers of a different character and meaning. Santander et al. [24] reported that the aqueous fraction of *P. alliacea* activated human dendritic cells (antigen-presenting cells that link innate and acquired immunity) via an NF-κB-dependent pathway, leading to upregulation of co-stimulatory molecules and pro-inflammatory cytokines. Immunostimulatory properties of *P. alliacea* are important; they indicate that aqueous fractions extracted from this plant could stimulate acquired immunity rather than suppress it. This aspect is clinically relevant; stimulation of DC activation is not recommended during the treatment of autoimmune or hypersensitivity disorders.

Another study on *P. alliacea* [25] investigated the impact of the compound DTS, a sulfurous substance typical of the plant. Instead of demonstrating neuroprotective activity, the authors revealed that the compound disrupted microtubule assembly and neurite outgrowth in neuroblastoma cells and inhibited MAP kinase tyrosine dephosphorylation. This finding has dual implications. First, it reveals the agent's neurotoxicity at certain doses and must be considered when evaluating its applicability in neurological diseases. Second, while a pathological phenomenon, the disturbance of microtubule and MAPK dynamics may, in some circumstances, contribute to antiproliferative effects, as exemplified by tumor models. In summary, although promising in other respects, DTS cannot yet be recommended for use in neurological diseases.

Biological Outcomes and Mechanistic Pathways

Table 3 presents a cross-study overview of key outcomes, mechanisms, and quality-appraisal findings. Across the three plants, converging evidence implicates several shared mechanistic axes.

**Table 3:** Biological outcomes and mechanistic pathways identified across included studies ROS: reactive oxygen species, NADPH: nicotinamide adenine dinucleotide phosphate, ICAM-1: intercellular adhesion molecule-1, TNF-α: tumor necrosis factor-alpha, PI3K/AKT: phosphoinositide 3-kinase/protein kinase B signaling pathway, NF-κB: nuclear factor kappa B, miR: microRNA, NSCs: neural stem cells, SIRT1: sirtuin 1, SCFAs: short-chain fatty acids, Th1/Th2: T-helper cell type 1/type 2, HO-1: heme oxygenase-1, MAPK: mitogen-activated protein kinase, DC: dendritic cell, BBB: blood-brain barrier, IL-1β: interleukin-1 beta, IL-8: interleukin-8, BRD4: bromodomain-containing protein 4, CNS: central nervous system

Authors (year)	Outcome category	Key outcomes	Mechanism	Key findings	Conclusion
Mahomoodally et al. 2012 [18]	Immunomodulatory	ROS production	NADPH oxidase inhibition	↓ ROS (40-65%)	Modest immunomodulatory effect
Sung et al. 2018 [19]	Immunomodulatory/anti-inflammatory	ICAM-1, TNF-α, leukocyte adhesion	PI3K/AKT/NF-κB via miR-221/222	↓ ICAM-1, ↓ inflammation	Strong anti-inflammatory effect
Hu et al. 2020 [20]	Neuroprotective/neurogenic	NSC differentiation, β-catenin	SIRT1 → β-catenin signaling	↑ Neuronal differentiation	Neuroregeneration potential
Zhang et al. 2025 [21]	Anti-inflammatory	Cytokines, SCFAs	Gut microbiota modulation	↑ SCFAs, ↓ inflammation	Prebiotic anti-inflammatory effect
Fachinan et al. 2017. [22]	Immunomodulatory	T-cell proliferation	Th1/Th2 modulation	Shift to Th2 immune phenotype	Immune-polarizing effect
Kim et al. 2018 [23]	Neuroprotective/antioxidant	ROS, apoptosis	HO-1 upregulation + MAPK inhibition	↓ ROS, ↓ apoptosis	Strong antioxidant/neuroprotective activity
Santander et al. 2012[24]	Immunomodulatory	Dendritic cell activation	NF-κB signaling	↑ Co-stimulatory molecules, ↑ cytokines	DC-mediated immune stimulation
Rosner et al. 2001 [25]	Neurotoxicity/mechanistic	Neurite outgrowth, MAPK	Microtubule disruption	↓ Neurite growth; MAPK inhibition	Safety concern at relevant concentrations
Mancini et al. 2018 [26]	Neuroprotective delivery	BBB permeability, antioxidant activity	Nanodelivery formulation	↑ BBB penetration without antioxidant loss	Drug delivery potential for CNS targeting
Chuang et al. 2020 [27]	Anti-inflammatory	IL-1β, IL-8	MAPK + caspase-1 inhibition	↓ Cytokines in vitro and in vivo	Effective anti-inflammatory effect
Deng et al. 2019 [28]	Immunomodulatory/immunotoxic	Cytokines, apoptosis	Dose-dependent modulation	Dual effects: cytotoxic at high doses	Dose caution required
Leivas et al. 2023 [29]	Anti-inflammatory/analgesic	Pain, leukocyte migration	Polysaccharide structure-function	↓ Nociception, ↓ leukocyte migration	Analgesic and anti-inflammatory potential
Wu et al. 2026 [30]	Immunomodulatory/epigenetic	Autoantibodies, cytokines	miR-146a/BRD4 axis regulation	↓ Autoimmunity markers	Epigenetic immunomodulation potential

Quality Appraisal Results

Tables 4-5 show the outcomes of the MMAT and JBI quality appraisals, respectively. The quality of methodology used in the selected articles was found to be moderate to high. However, some areas needed improvement.

**Table 4:** MMAT quality appraisal (in vitro and mixed-design studies) Risk of bias was qualitatively categorized as low or moderate based on methodological transparency, sample reporting, control measures, and the reproducibility of experimental procedures. Overall quality ratings were assigned based on a cumulative assessment of study design, methodological rigor, outcome assessment, interpretation of findings, and reproducibility. Yes: criterion adequately fulfilled, Partial: criterion partially fulfilled or incompletely reported

Authors (year)	Clear research question	Appropriate design	Methodological rigor	Outcome measurement	Risk of bias	Data interpretation	Reproducibility	Overall quality
Mahomoodally et al. 2012 [18]	Yes	Yes	Moderate	Yes	Moderate	Yes	Moderate	Moderate
Sung et al. 2018 [19]	Yes	Yes	Yes	Yes	Low	Yes	Yes	High
Zhang et al.2025 [21]	Yes	Yes	Yes	Yes	Moderate	Yes	Partial	Moderate-High
Fachinan et al. 2017 [22]	Yes	Yes	Yes	Yes	Moderate	Yes	Partial	Moderate-High
Kim et al. 2018 [23]	Yes	Yes	Yes	Yes	Moderate	Yes	Partial	High
Santander et al. 2012 [24]	Yes	Yes	Yes	Yes	Moderate	Yes	Partial	High
Rösner et al. 2001 [25]	Yes	Yes	Yes	Yes	Moderate	Yes	Partial	High
Mancini et al. 2018 [26]	Yes	Yes	Yes	Yes	Moderate	Yes	Partial	High
Leivas et al. 2023 [29]	Yes	Yes	Moderate	Yes	Moderate	Yes	Partial	Moderate

**Table 5:** JBI quality appraisal (animal and high-level experimental studies) “Yes” indicates that the methodological criterion was adequately reported or fulfilled, whereas “Partial” and “Unclear” indicate incomplete or insufficient reporting. Overall quality ratings were based on methodological rigor, the reliability of outcome assessment, statistical analysis, and the depth of mechanistic investigation. MCAO: middle cerebral artery occlusion, WB: Western blotting, IF: immunofluorescence

Authors (year)	Randomization	Control group	Blinding	Outcome measurement reliability	Statistical analysis	Mechanistic depth	Overall quality
Hu et al. 2020 [20]	Yes	Yes (Sham + MCAO)	Partial	High (WB, IF, SIRT1)	Appropriate	Very High	High
Chuang et al. 2020 [27]	Unclear	Yes	Unclear	Moderate	Yes	High	Moderate
Deng et al. 2019 [28]	Yes	Yes	Moderate	Low (sample size unclear)	Yes	Moderate	Moderate

Discussion

Principal Findings

This systematic review summarizes the mechanisms underlying the neuroprotective and immunomodulatory effects of three Caribbean medicinal plants. The convergence in biological activity included reductions in oxidative stress and pro-inflammatory cytokine effects, as well as involvement in canonical inflammatory signaling pathways, such as NF-κB, MAPK, and SIRT1/β-catenin. Among the neuroprotective effects, the prevention of neuronal apoptosis, NSC-induced neurogenesis, and improved delivery into the brain parenchyma achieved using nanoformulation approaches with *A. muricata* are the most notable findings for *M. charantia*. New mechanistic insights into epigenetic regulation by miRNAs and gut-immune axis manipulation by SCFA-producing polysaccharides can be considered an advantage over the conventional phytochemical approach for understanding the underlying mechanisms.

Mechanistic Patterns and Their Significance

The PI3K/AKT/NF-κB pathway is the most commonly reported in the literature for both *M. charantia* and *P. alliacea* [19,24,27]. Inflammation regulation and cellular survival signaling are controlled by the PI3K/AKT/NF-κB pathway, which also shows modulatory potential of several plant-based products, as reported in the broader literature on phytopharmacology [5]. The data from our review align with existing literature, indicating that NF-κB repression is a common downstream mechanism by which various plant constituents exert their biological effects; this applies to both polysaccharide- and peptide-rich extracts and to phenolic fractions.

The SIRT1/β-catenin axis described by Hu et al. [20] was particularly significant in that it was linked with NSCs undergoing differentiation into neurons rather than protecting them against injury. This opens up new opportunities in neuroregenerative therapy rather than neuroprotection alone. This finding was based solely on a single paper; however, each group had a relatively small sample size (n = 6). This study is therefore exploratory and should be interpreted accordingly.

The gut microbiota-based anti-inflammatory mechanism reported by Zhang et al. [21] is one of the insights into pharmacology that often go unnoticed when investigating traditional medicine. The fermentation of polysaccharides generates SCFAs and suppresses cytokine activity, supporting the classification of these compounds as potential microbiome-based therapeutics. The fact that the mechanism discussed above is systemic rather than local suggests broader prospects for the oral application of such plant fractions. As reported in recent studies on *M. charantia* polysaccharides [12], structural variability suggests a possible dependence of prebiotic properties on the fraction and degree of polymerization.

Strengths of Evidence and Limitations of Individual Studies

Using the results of quality appraisal to interpret the evidence provides a clear differentiation in the degree of confidence that can be placed in various findings. The findings reported by Hu et al. [20] have been assigned a high score on the JBI scale. These findings have high validity given the randomization, proper sham and experimental controls, and high-quality mechanistic validation via Western blot and immunofluorescence. Hence, the neuroprotective and neurogenic findings are more likely to be true. On the other hand, Sung et al. [19] present a comprehensive mechanism linking miRNA to inflammatory adhesion molecules, and their animal and cell model studies provide sufficient evidence of inhibition of the PI3K/AKT/NF-κB pathway.

On the other hand, studies rated moderately need to be interpreted with greater care. For example, Chuang et al. [27] reported unclear processes of randomization and blinding, although the studies were in vivo. Such a situation makes it possible for performance and detection biases to exist. Similarly, in Deng et al. [28], the number of subjects included was unclear, reducing the accuracy of their dose-response curve. The study by Rösner et al. [25] is mechanically precise; however, it predates current research standards and lacks validation in an in vivo neurological model. Thus, their findings on the neurotoxicity of DTS suggest that caution is warranted without ruling out the potential therapeutic role of *P. alliacea* altogether.

A few issues cut across several studies included in this review. Since there is no established procedure for extracting, even studies using seemingly the same plant parts, such as *M. charantia* fruit, could be evaluating entirely different compounds. Moreover, reliance on in vitro and small animal testing implies that the clinical significance of these mechanisms, involving the absorption, distribution, metabolism, and excretion of these bioactive agents, is still not known.

Comparative and Translational Perspective

The results of the analysis of these herbs align with the findings of most scientific studies conducted in animal models. Studies demonstrating the ability of *M. charantia* and *A. muricata* to reduce oxidative stress by activating Nrf2/HO-1 signaling pathways [30-32] and regulating NF-κB cytokines [33-36] have been conducted independently by various scientists and across model organisms, supporting the consistency of the results reported in this review. Neuroprotective effects of *P. alliacea*, as revealed in recent studies on its protection against scopolamine-induced amnesia and its inhibition of amyloid-β protein aggregation [33], provide further insight into the plant's CNS-related effects beyond the limited information gathered in the present study [37,38].

It should be noted that a considerable translational gap remains between the complex mechanisms elucidated in preclinical studies and their potential clinical applications. The only human clinical study on *M. charantia* and glycemia [39,40] does not investigate its neuroprotective or immunological effects; moreover, no clinical studies have specifically evaluated these outcomes. This remains a major obstacle to clinical translation. In other words, although the mechanism by which these drugs exert their effects is now relatively well known, their efficacy, toxicity, and appropriate dosages remain unknown in humans. As noted by Mancini et al. [26], one potential strategy for developing such drugs would be utilizing nanoformulations, thereby increasing their bioavailability in the central nervous system.

Policy and Public Health Implications

However, the applicability of this study extends beyond pharmacological research to public health policy and health system management. Both the WHO Traditional Medicine Strategy and SDG 3 emphasize the need to develop evidence-based integration of traditional medicine practices, and the findings of this study, being purely preclinical, can serve as part of such an evidence base. In particular, in the context of the Caribbean region, where these plants are widely used, the systematic characterization of the preclinical data serves an important role in helping the health officials formulate guidelines regarding the traditional use of these plants, highlighting their benefits as well as possible risks (especially about toxic effects that occur in the case of overdose of α-momorcharin and DTS) [28,25,41].

It is necessary to stress that while the review may be connected to disaster medicine frameworks, including the Sendai Framework for Disaster Risk Reduction, this connection should not be overstated. This study presents no evidence of the effectiveness of these plants in post-disaster scenarios. However, the policy implication is not nearly as ambitious but still significant: in environments where the supply of pharmaceuticals is compromised, the availability of safe, effective plant medicines that can be produced locally and backed by preclinical evidence is genuinely advantageous from a community health standpoint. Formalizing such an advantage through quality control of plant-derived substances, training community health workers, and sound public health advice is a sensible policy path to pursue, based on current evidence, insofar as clinical trials continue.

## Conclusions

The systematic analysis of 13 preclinical studies has revealed that the fruits of *M. charantia*, the seeds of *A. muricata*, and the roots of *P. alliacea* exhibit significant neuroprotective and immunomodulatory properties in experimental models, acting via known cellular mechanisms like the PI3K/AKT/NF-κB pathway, MAPK signaling, Nrf2/HO-1 induction, SIRT1/β-catenin regulation, and epigenetics via miRNAs. Of these three plants, *M. charantia* has the most developed and thorough evidence base, while *A. muricata* and *P. alliacea* have less evidence and warrant additional primary research. However, although this body of evidence appears promising, it is exclusively preclinical. Various issues in methodological rigor, such as heterogeneous extracts used, inconsistent reporting of sample sizes, single-reviewer study screening, and a lack of clinically relevant data, have implications that limit the confidence that can be placed in the individual results obtained, making direct application difficult as well. Dose-specific toxicity with certain extracts requires special care and calls for pharmacokinetic analysis and extensive dose-finding experiments. Further research needs to emphasize the design and conduct of appropriately designed clinical trials to assess safety, tolerability, and dose-response effects in human subjects; the standardization of extracts to ensure cross-comparability across studies; and the exploration of lesser-known mechanisms and substances, especially *A. muricata* and *P. alliacea*. Multidisciplinary efforts from pharmacology, ethnobotany, and public health are necessary to translate this preclinical evidence into applicable contributions to healthcare in the Caribbean and beyond.
